# Maximum Plant Uptakes for Water, Nutrients, and Oxygen Are Not Always Met by Irrigation Rate and Distribution in Water-based Cultivation Systems

**DOI:** 10.3389/fpls.2017.00562

**Published:** 2017-04-11

**Authors:** Chris Blok, Brian E. Jackson, Xianfeng Guo, Pieter H. B. de Visser, Leo F. M. Marcelis

**Affiliations:** ^1^Wageningen Plant Research, Wageningen University and ResearchWageningen, Netherlands; ^2^Department of Horticultural Science, North Carolina State University, RaleighNC, USA; ^3^College of Forestry, Shandong Agricultural UniversityTai’an, China; ^4^Horticulture and Product Physiology, Wageningen University and ResearchWageningen, Netherlands

**Keywords:** aeroponic mist, *Chrysanthemum morifolium*, hydroponic, deep flow technique, nutrient flow technique, raft culture, rooting media, water culture

## Abstract

Growing on rooting media other than soils *in situ* -i.e., substrate-based growing- allows for higher yields than soil-based growing as transport rates of water, nutrients, and oxygen in substrate surpass those in soil. Possibly water-based growing allows for even higher yields as transport rates of water and nutrients in water surpass those in substrate, even though the transport of oxygen may be more complex. Transport rates can only limit growth when they are below a rate corresponding to maximum plant uptake. Our first objective was to compare *Chrysanthemum* growth performance for three water-based growing systems with different irrigation. We compared; multi-point irrigation into a pond (DeepFlow); one-point irrigation resulting in a thin film of running water (NutrientFlow) and multi-point irrigation as droplets through air (Aeroponic). Second objective was to compare press pots as propagation medium with nutrient solution as propagation medium. The comparison included DeepFlow water-rooted cuttings with either the stem 1 cm into the nutrient solution or with the stem 1 cm above the nutrient solution. Measurements included fresh weight, dry weight, length, water supply, nutrient supply, and oxygen levels. To account for differences in radiation sum received, crop performance was evaluated with Radiation Use Efficiency (RUE) expressed as dry weight over sum of Photosynthetically Active Radiation. The reference, DeepFlow with substrate-based propagation, showed the highest RUE, even while the oxygen supply provided by irrigation was potentially growth limiting. DeepFlow with water-based propagation showed 15–17% lower RUEs than the reference. NutrientFlow showed 8% lower RUE than the reference, in combination with potentially limiting irrigation supply of nutrients and oxygen. Aeroponic showed RUE levels similar to the reference and Aeroponic had non-limiting irrigation supply of water, nutrients, and oxygen. Water-based propagation affected the subsequent cultivation in the DeepFlow negatively compared to substrate-based propagation. Water-based propagation resulted in frequent transient discolorations after transplanting in all cultivation systems, indicating a factor, other than irrigation supply of water, nutrients, and oxygen, influencing plant uptake. Plant uptake rates for water, nutrients, and oxygen are offered as a more fundamental way to compare and improve growing systems.

## Introduction

Greenhouse horticulture delivers the world’s highest ever agricultural resource use efficiencies for water and nutrients (Stanghellini, 2014). Water use efficiency is highly relevant as agricultural use is contributing significantly to the depletion of the world’s scarce fresh water sources (FAO, 2002). Nutrient use efficiency is relevant as the diffuse emission of fertilizers into the environment is highly disruptive for the world’s ecology (Foley et al., 2011; Bindraban et al., 2015). In greenhouse horticulture and some high value outdoor crops, the combination of soilless growing and recirculation of drainage solution in recirculating soilless systems is the most effective way to reduce emissions of water, nutrients, and also plant protection products into the environment (Grewal et al., 2011; Stanghellini, 2014). Recirculating soilless systems are successfully used in global commercial production of greenhouse crops such as tomato, pepper, and roses (González Céspedes et al., 2004). For large scale *chrysanthemum* growing, however, no recirculating soilless systems have so far been successfully applied. The financial investments in substrate- or water-based growing systems are not met by a sufficient advantage in yield per unit area, which raises the question what yield advantage is to be expected from growing in either substrate or water over soil-based growing? Such yield advantages are brought about by: (1) Transport rates of water and nutrients in substrate are higher than in soil. Combined water and nutrient supply in soil-based growing requires large irrigation intervals, to allow for sufficient oxygen supply through temporarily air filled pores in between irrigation cycles (Evans et al., 2009; Assouline et al., 2012). In substrates, however, larger amounts of pore space (porosity) allow for more frequent irrigation than in soil-based growing (Assouline et al., 2012). Increasing the irrigation frequency from 1 to 30 cycles a day increased fresh (FW) and dry weight (DW) growth of *Chrysanthemum* with 10–30% (Buwalda and Kim, 1994; Silber et al., 2003, 2005; Xu et al., 2004). Irrigation frequency is therefore a key management factor in maintaining a flow of water and nutrients toward the roots which is large enough to surpass the plant water uptake (Raviv et al., 1999; De Swaef et al., 2011) and nutrient uptake (Silber et al., 2003; Xu et al., 2004). Both, transport rates of water and of nutrients, are highly dependent on substrate characteristics including water filled pore space and pore geometry (Allaire et al., 1994; Raviv et al., 1999; Caron et al., 2002; De Swaef et al., 2011). The influence of irrigation volume, substrate volume, and plant density on water and nutrient uptake was modeled and validated (Bar-Tal et al., 1993). (2) Transport rates of oxygen in substrate are higher than in soil. Oxygen transport rates in soil and substrate-based growing are dependent on air content and pore geometry (Allaire et al., 1996; Caron and Nkongolo, 2004; Dresbøll and Thorup-Kristensen, 2012). As water content, including dissolved nutrients, and air content are inversely related, this means both must be optimized concomitantly. Effective substrates typically have total porosities of over 80% compared to typical soil porosities of below 40% (Raviv and Lieth, 2008). The higher porosities of substrates ensure there are enough water filled pores for water and nutrient transport as well as enough air filled pores for oxygen transport. Fertigation systems and substrates both differ in their ability to at all times keep up with the highly fluctuating plant requirements for water, nutrients, and oxygen (Naasz et al., 2005). (3) Root penetration in compressed and uncompressed substrates is faster than in soil. However, root penetration in compressed substrate is slower than in uncompressed substrate, as has been shown for press pots of compacted black peat mixes (Kämpf et al., 1999) which are used for the propagation of *chrysanthemum*, lettuce, and *Eustoma*. The penetrability of press pots compared to uncompressed substrate decreased growth during a 14-day propagation stage by 5 days (Hansen, 1999; Arancon et al., 2015).

The evidence of the above transport rate dependent influences on yield now allows us to reflect: (1) Transport rates are expected to affect growth only when transport rates are below the maximum plant uptake. The maximum plant uptake is defined as the highest possible uptake to be expected under optimum commercial circumstances of light, carbon dioxide, temperature, water, nutrients, and oxygen. (2) Transport rate differences explain why different substrates require different irrigation strategies for optimum production. Comparisons of crop growth on different substrates are often of limited value since results not only change with substrate material properties but also with irrigation strategy, substrate height, substrate volume per unit area, and evapotranspiration. Our ambition is to leave the empirical direct comparison of growth on substrate systems in favor of comparing transport rates to maximum plant uptakes. Maximum plant uptakes can be linked to climate physical and plant physiological properties of more general validity.

If particle shape and total particle volume reduce transport rates in substrate-based growing as is discussed, the absence of particles will allow for higher transport rates in all water-based growing systems. Propagation in water followed by cultivation in water might also avoid common problems with root development when transplanting from propagation medium into cultivation medium (Arancon et al., 2015). In water-based cultivation (i.e., excluding propagation), three main irrigation systems are (1) a multi-point supply in Deep Flow Technique (DFT); (2) a one-point supply in Nutrient Flow Technique (NFT); and (3) a multi-point supply in aeroponics. (a) DFT, also called floating boards or rafts, is a method for growing plants on reservoirs of 5–30 cm nutrient solution depth in broad field (Shi et al., 2007). Irrigation solution is distributed by either a one-point supply creating water movement in the pond or -as we choose- by multi-point supply from the bottom of the pond. The maximum uptake of oxygen by the roots may not be met as oxygen has to be transported to the root surface of plants in every plant position in the pond including corner positions. (b) NFT is a method for growing plants in 0–1 cm running solution in gullies (Cooper, 1975; Pardossi et al., 2002). Irrigation solution is distributed by a one-point supply at the highest position of the gully. The irrigation supply should ensure that the last plant in a gully still receives enough water, nutrients and dissolved oxygen. (c) Aeroponics, or mist systems, is a method for growing plants on nutrient solution sprayed on the roots in an air filled chamber (Soffer et al., 1991; Zhao et al., 2010). Irrigation solution is distributed by a multi-point nozzle system. For an aeroponic system, with a supposed optimal supply of oxygen through air, the supply could be as low as the maximum plant uptake for water or nutrients allows.

Each of the water-based growing systems thus has distinct consequences for the transport rates of water, nutrients, and oxygen, despite the general advantage of the absence of transport rate slowing particles over substrate-based growing systems. The first objective of this work was to compare *chrysanthemum* growth performance for three water-based growing systems with different irrigation systems. The second objective was to compare press pots as propagation medium with a nutrient solution as propagation medium for *chrysanthemums*. Our hypotheses were: (1) Production of the growing systems would be similar with a possibly more homogeneous growth in aeroponics over DFT and NFT, as oxygen supply in aeroponics is expected to be more homogeneous than in DFT and nutrient supply more homogeneous than in NFT; (2) Production in a water-based cultivation system would be higher when using cuttings from water-based propagation than when using cuttings from substrate-based propagation as water-based propagation followed by water-based cultivation would avoid sudden changes in transport rates of water, nutrients, and oxygen.

## Materials and Methods

Three consecutive crop cycles were conducted in a 12.0 m × 12.8 m glasshouse compartment of the Venlo multispan facility at Bleiswijk research station of Wageningen University and Research, Netherlands (Latitude 52°1′ N, Longitude 4°3′ E). The compartment was equipped with energy screen, light screen, black-out screen, and 180 μmol m^-2^ s^-1^ PAR (Photosynthetically Active Radiation) supplementary light from high-pressure sodium lamps (SON-T, Philips, Best, Netherlands).

### Propagation

Substrate-rooted *chrysanthemum* cuttings as well as bare cuttings were obtained from a commercial propagator (*Chrysanthemum morifolium* [Indica group] ‘Euro’, Dekker *Chrysanthemum*, Hensdijk, Netherlands). All cuttings were pre-treated with rooting powder containing 2.5 mg g^-1^ indole butyric acid (IBA). At the propagator, bare cuttings were stuck in 5 cm × 5 cm × 5 cm press pots of a mix of milled peat and frozen black peat and kept at 20°C day and night. Cuttings grew for 14 days at a density of 400 per m^2^ with 108 μmol m^-2^ s^-1^ PAR supplementary light from high-pressure sodium lamps, prior to delivery as substrate-rooted cuttings. At the Bleiswijk research station bare cuttings were stuck in a perforated floating Styrofoam board on a nutrient solution of 26°C and kept at 26°C air temperature both day and night. Relative humidity was kept at 90–95% and CO_2_ level was 700 ppm. Cuttings grew for 5 days at a density of 1200 per m^2^ with 180 μmol m^-2^ s^-1^ PAR supplementary light from high-pressure sodium lamps, prior to transplanting as water-rooted cuttings.

### Cultivation

Transplanting dates were April 29, August 20, and November 25, 2010. Plant density was 57 plants per m^2^ (**Table 1**). The day and night temperature were set at 20 and 19.5°C and the relative humidity was 75–95%. The CO_2_ level was maintained at 1000 ppm except for the first 4 days at 700 ppm. 0.2 L m^-2^ of a plant growth controller, used to reduce length growth, *N*-Dimethylamino succinamic acid (daminozide, Alar85, Certis), was sprayed with concentration 2–4 g L^-1^, 4 g L^-1^ (0.4–0.8 g m^-2^) at Day After Planting 29 (DAP 29) and DAP 36 for all treatments but with the lower dose for the smaller plants on DAP 29. Plants received a “long day” treatment of 16 h of light partly supplied as supplementary light. When 12 leaf pairs had formed, the plants received a “short day” treatment of 12 h of light per day partly by using the black-out screen.

**Table 1:** Plant density and time details of three *chrysanthemum* cultivation cycles.

Parameter	Unit	Cycle 1	Cycle 2	Cycle 3
Duration of water-based propagation	Days	5	5	5
Plant density water-based propagation	m^-2^	1200	1200	1200
Duration of press pot propagation	Days	14	14	14
Density press pot propagation	m^-2^	400	400	400
Planting date	Date	29-4-2010	24-8-2010	25-11-2010
Start date short day treatment	Date	17-5-2010	11-9-2010	12-12-2010
Harvesting date	Date	5-7-2010	2-11-2010	10-2-2011
Duration of long day period	Days	18	20	17
Duration of short day period	Days	49	52	60
Duration of total in cultivation	Days	67	72	77
Duration of propagation + cultivation	Days	81	86	91
Plant density	m^-2^	57	57	57

*Start and stop dates, duration, and plant density are given for water-based propagation, press pot propagation, long day cultivation, and short day cultivation periods for three crop cycles. Duration in days after planting for propagation and in days after transplanting for cultivation.*

### Treatments

**FIGURE 1:**
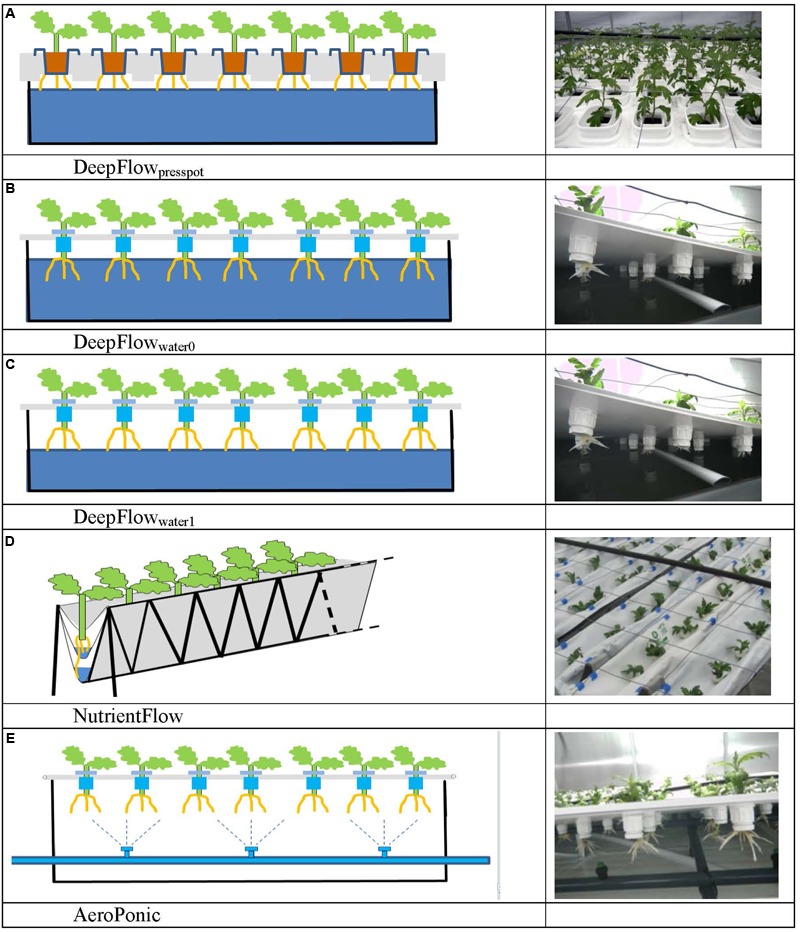
**Schedules and photos of five treatments.** DeepFlow_presspot_, Deep Flow System with press pots, i.e., substrate-rooted cuttings **(A)**. DeepFlow_water0_, Deep Flow System water-rooted cuttings immersed in the nutrient solution surface **(B)**. DeepFlow_water1_, Deep Flow System, with water-rooted cuttings and solution surface at 1 cm below the basal end of the cuttings **(C)**. NutrientFlow, Nutrient Flow System **(D)**. AeroPonic, Aeroponic Mist System **(E)**. NutrientFlow and AeroPonic both used water-rooted cuttings.

Five treatments based on the three main groups of water-based cultivation systems were tested during three crop cycles in 2010–2011 (**Figure 1** and **Table 1**). The number of plots per treatment was 6 (cycle 1), 14 (cycle 2), and 16 (cycle 3). This allowed us to test variants of the DFT system in cycles 2 and 3. Furthermore we abandoned the NFT system after cycle 2 due to technical impracticalities. The resulting unbalanced design was analyzed as unbalanced block design using pair wise *t*-tests.

Cultivation systems were built on ebb-and-flow benches 180 cm × 120 cm × 10 cm and each treatment used a combined light impermeable cover/plant support above the 180 cm × 120 cm bench area. The treatments were: (1) A Deep Flow System with plants rooted in press pots (DeepFlow_presspot_; **Figure 1A**). Light impermeable cover/plant support was provided by 120 cm × 40 cm × 3.5 cm Styrofoam boards (Dry Hydroponic system, Cultivation, Schipluiden, Netherlands), cut to seamlessly cover the 180 × 120 benches. The Styrofoam boards floated on the surface of a pond of nutrient solution with a depth of 6 cm under the boards. Substrate-rooted cuttings were planted in 10 cm × 10 cm polyethylene clamps that fitted into holes in the Styrofoam boards. The clamps held the press pots 1–2 cm above the nutrient solution but prevented press pots from touching the nutrient solution. Substrate-rooted cuttings were used of about 8 cm long with four leaf pairs and a root mass of 0.2 g with 20 roots of 6 cm. DeepFlow_presspot_ was used in all three growing cycles. The nutrient solution in the pond was stirred with two irrigation cycles of 5 min per hour by the turbulence caused by jets of nutrient solution from a multi-point perforated tube in the nutrient solution (**Table 2**). An overflow kept pond levels stable. (2) A Deep Flow System with water-rooted cuttings and the basal end of the cuttings immersed in the nutrient solution (DeepFlow_water0_; **Figure 1B**). Light impermeable cover/plant support was provided by a rigid 180 cm × 120 cm white coated high density fiber board (HDF). Water-rooted cuttings were stuck into 20 mm diameter rubber caps which covered small poly ethylene sockets (16 mm in diameter and 32 mm in height) inserted in the HDF board. The HDF board was suspended 2 cm above the surface of a pond of nutrient solution with a depth of 6 cm. Water-rooted cuttings were 6 cm long with three leaf pairs and no roots longer than 6 mm. Cuttings were about 1 cm deep into the nutrient solution. DeepFlow_water0_ was used in growing cycles 2 and 3. Irrigation was arranged as for DeepFlow_presspot_. (3) A Deep Flow System with water-rooted cuttings and the solution surface at 1 cm below the basal end of the cutting (DeepFlow_water1_; **Figure 1C**). Identical to DeepFlow_water0_ but pond with a depth of 4 cm leaving 1 cm of air between the basal end of the cutting and the solution surface. DeepFlow_water1_ was used in growing cycle 3 only. Irrigation was arranged as for DeepFlow_presspot_. (4) A Nutrient Flow System (NutrientFlow; **Figure 1D**). Light impermeable cover/plant support was provided by a black and white poly ethylene top foil in single plant rows of 14 cm wide and 120 cm long. The top foil and two lower layers of foil under the top foil hung in an 8 cm wide V-shape with the layers 4 cm apart and the top layer V-shape 4 cm deep. The foil layers were held in position by a 20 cm high galvanized iron wire structure on a 2 cm m^-1^ slope (foil and supports; NGS New Growing System, Almeria, Spain). The slope allowed the nutrient solution in the row to drain down to the lower end. Nutrient solution was supplied through 2 L h^-1^ drippers with a one-point emission at the highest point of each row, 10 plants per row and fertigation set at six irrigation cycles of 30 s per hour (**Table 2**). Water-rooted cuttings were stuck in 20 mm diameter rubber caps and then in slits in the top foil, with the caps preventing the cuttings from sinking deeper into the slit. Ebb-and-flow benches held 12 rows of plants. NutrientFlow was used in growing cycles 1 and 2. (5) An Aeroponic Mist System (AeroPonic; **Figure 1E**). Light impermeable cover/plant support was provided by a rigid 180 cm × 120 cm HDF board. Water-rooted cuttings were stuck into 20 mm diameter rubber caps which covered small poly ethylene sockets (16 mm in diameter and 32 mm in height) inserted in the HDF board. The HDF board was suspended 21.5 cm above the surface of a bench with HDF side boards all around. In the resulting root chamber, a multi-point irrigation was supplied by eight 100 L h^-1^ nozzles per bench. The nozzles sprayed nutrient solution onto the basal part of the cuttings during 12 irrigation cycles of 30 s per hour (**Table 2**). AeroPonic was used in all three growing cycles.

**Table 2:** Cultivation system parameters on root available volume, resident water volume, fertigation cycle number, fertigation duration, supply interval, and supply rate for different systems.

Parameter	Unit	DeepFlow _presspot/water0/water1_	NutrientFlow	AeroPonic
Root available space	L m^-2^	60	100^∗^	300^∗^
Resident water	L m^-2^	60^∗∗^	0.5–1.5	0.5–1.5
Fertigation cycles	h^-1^	2	6	12
Irrigation duration	min cycle^-1^	5	0.5	0.5
Irrigation duration	min h^-1^	10	3	6
Emitter capacity	L h^-1^	2	2	100
Emitters per m^2^	m^-2^	20	7	4
Capacity per m^2^	L m^-2^ h^-1^	40	14	400
Irrigation rate	L m^-2^ min^-1^	0.67	0.23	6.67
Irrigation supply	L m^-2^ cycle^-1^	3.33	0.12	3.33
Irrigation supply	L m^-2^ h^-1^	6.67	0.70	40
Irrigation supply^∗∗∗^	L m^-2^ d^-1^	80	8.4	480

*DeepFlow_*presspot*_, Deep Flow System with press pots, i.e., cuttings substrate-rooted cuttings. DeepFlow_*water0*_, Deep Flow System with water-rooted cuttings immersed in the nutrient solution surface. DeepFlow_*water1*_, Deep Flow System with water-rooted cuttings solution surface at 1 cm below the basal end of the cuttings. NutrientFlow, Nutrient Flow System; AeroPonic, Aeroponic Mist System. NutrientFlow and AeroPonic both used water-rooted cuttings.*

*^∗^Mostly air filled space.*

*^∗∗^40 L m^-2^ for the DeepFlow_*water1*_ system with 4 cm of resident water.*

*^∗∗∗^12-h day.*

### Maximum Uptake Rates

Maximum plant uptakes of water, nutrients, and oxygen, were used as criteria to evaluate the irrigation supply of the growing systems. The maximum plant uptakes expressed per hour were based on general plant physics and experimental data. (A) Using general plant physics we found (1) Plant water uptake will at maximum equal the amount of incident radiation energy (Steppe et al., 2008; Janka et al., 2016). The maximum inside radiation level is 500 W m^-2^ which is 1.8 MJ m^-2^ h^-1^. Transpiration of 25°C water requires a heat of evaporation of 2.4 MJ L^-1^. Dividing the maximum inside radiation by 2.4 MJ L^-1^ renders a maximum water uptake of 0.70 L m^-2^ h^-1^. (2) Plant nutrient uptake corresponds with dry mass formation. Dry mass formation is described with light use efficiency in g dry mass per unit PAR light. In our experiment the maximum inside PAR radiation level was 0.97 MJ m^-2^ h^-1^. The light use efficiency of *chrysanthemum* is reported as 6.0 g MJ^-1^ for winter and 1.0 g MJ^-1^ for summer (Heuvelink et al., 2002). Multiplying maximum radiation and light use efficiency renders 1–2 g m^-2^ h^-1^ of dry mass production. Per g dry mass we expect 3.5 meq of nutrients (Sonneveld and Voogt, 2009) which is 3.5 to 7.0 meq for 1–2 g m^-2^ h^-1^ of dry mass. The resulting EC in 0.70 L m^-2^ h^-1^ would be 0.5–1.0 dS m^-1^. The concentration of ions (meq L^-1^) in a solution can roughly be estimated by multiplying the EC (dS m^-1^) by 10. The maximum nutrient uptake is therefore calculated as 0.7^∗^10 which is 7 meq L^-1^. (3) Plant oxygen uptake will at maximum be the amount taken in by the total root mass times the maximum uptake per unit mass of roots. For a closed canopy crop the root FW was taken as 1.0 kg m^-2^ and the maximum oxygen uptake was taken as 0.5–2.0 mg g(FW) h^-1^ for roots of tomato, rose, and cucumber as derived from (Morard et al., 2000; Bar-Yosef and Lieth, 2013; Blok and Gérard, 2013). The maximum plant uptake is therefore calculated as 2.0 g m^-2^ h^-1^, which is 62.5 mmol m^-2^ h^-1^. The maximum plant uptake of oxygen can also be expressed in liters of nutrient solution supplied by using a maximum oxygen level in a solution of 8.1 mg L^-1^ (at 25°C, 100 kPa air pressure and 3.0 dS m^-1^) which is about 0.25 mmol L^-1^ oxygen. The maximum plant uptake of 62.5 mmol m^-2^ h^-1^ therefore corresponds with 250 L m^-2^ h^-1^. (B) Using experimental data we found: (1) Plant water uptake was reported to be 0.7 L h^-1^ m^-2^ around noon on a summer day for *chrysanthemum* (Voogt et al., 2000; Janka et al., 2013); 0.7 L h^-1^ m^-2^ around noon on a summer day for rose (Baas and van Rijssel, 2006; Incrocci et al., 2014); and 0.5 L h^-1^ m^-2^ around noon in early April for tomato (Nederhoff and De Graaf, 1993; Klaring and Zude, 2009). (2) Plant nutrient uptake was reported to be 0.7 L h^-1^ m^-2^ with a plant uptake EC of 1.5 dS m^-1^ for *chrysanthemum* (Yoon et al., 2000a,b; Shima et al., 2002; Fernandes et al., 2012; Kaplan et al., 2016) which we calculated to a maximum nutrient uptake of 10 meq h^-1^ m^-2^; or a plant uptake EC of 1.0 dS m^-1^ for radish and tomato (Marcelis and Van Hooijdonk, 1999; Sonneveld and Voogt, 2009). (3) Plant oxygen uptake was reported mostly as uptake per gram of fresh root mass which does not allow an approach different from the one already shown.

Combining plant physics and experimental data and using the higher of the two approaches, in our study maximum water uptake was set at 0.70 L m^-2^ h^-1^; maximum nutrient uptake was set at 10 meq m^-2^ h^-1^ and maximum oxygen uptake was set at 62.5 mmol m^-2^ h^-1^.

The maximum plant uptakes found depend on a set of conditions and growing practices which limit the validity. Data are valid for greenhouse crops under near optimum conditions for light, temperature, and supply of water, nutrients, and carbon dioxide. It is, however, possible to find lower or higher maximum uptake rates with less common conditions. Conditions to be considered are air temperature, vapor pressure deficit, wind speed, carbon dioxide concentration, incident radiation (natural or artificial) crop management, pest, and diseases.

### Water and Nutrients

Water from a rain water collection basin with EC < 0.2 dS m^-1^ and pH 5.0 was used to prepare a nutrient solution for *chrysanthemum* (**Table 3**). The initial nutrient solution contained 1.58 mmol L^-1^ of ammonium nitrate (NH_4_NO_3_), 2.45 mmol L^-1^ of calcium nitrate [Ca(NO_3_)_2_], 0.90 mmol L^-1^ of monopotassium phosphate (KH_2_PO_4_), 1.35 mmol L^-1^ of magnesium sulfate (MgSO_4_), 3.30 mmol L^-1^ of potassium nitrate (KNO_3_), 0.25 mmol L^-1^ of potassium bicarbonate (KHCO_3_) and 1.60 mmol L^-1^ of potassium sulfate (K_2_SO_4_). Trace element levels were 67 μmol L^-1^ iron as EDDHA 6% iron chelate, 11.3 μmol L^-1^ manganese sulfate (MnSO_4_), 5.6 μmol L^-1^ zinc sulfate (ZnSO_4_), 22.5 μmol L^-1^ borax (Na_2_B_4_O_7_), 1.1 μmol L^-1^ copper sulfate (CuSO_4_), and 1.1 μmol L^-1^ sodium molybdate (Na_2_MoO_4_)_._ The nutrient solution was supplied to 400 L storage tanks under each bench, and each bench had an independent automated dosing system. For all systems drainage solution was collected via an overflow into the nutrient solution storage tanks under the benches, creating full recirculation of water and nutrients. The storage tanks were automatically replenished when the nutrient solution level was below half the capacity of the storage tank and the amount was recorded. The solution was changed from a vegetative to a generative solution on the moment the short day phase started, which was after 18 (cycle 1), 20 (cycle 2), and 17 (cycle 3) days of long day phase. Drainage of individual irrigation cycles was not recorded. EC and pH in the nutrient solution were manually checked and adjusted weekly to be kept at pH 5.5 and EC 1.8. The EC in cycle 1 dropped and was, 14 days after planting in cycle 1, raised to 2.4 dS m^-1^ which level was maintained. Thereafter, the concentration of all individual macro and micro nutrients in the storage tanks under one bench per treatment was checked every 14 days with Ion Coupled Plasma analysis by a commercial laboratory (Groen Agro Consult, Delft, Netherlands). The analysis results were compared with standardized target values (De Kreij et al., 1999). When individual element levels deviated over 20% from the standardized target values the solution added to the storage tank was adjusted. Uptake rates between two moments, A and B, were calculated by (a) multiplying concentration and volume at moments A and B into quantity A and quantity B; (b) subtracting quantity A minus quantity B to find the quantity of uptake; (c) dividing the quantity of uptake by area and time elapsed between moments A and B to find uptake rate as quantity per unit area per unit time.

**Table 3:** *Chrysanthemum* nutrient solutions applied for vegetative (Veg.) or long day period and generative (Gen.) or short day phases, before and after (EC+) increase of the EC to better meet plant uptake.

	EC, pH, and major elements									
Recipe	EC	pH	NH_4_	K	Ca	Mg	NO_3_	SO_4_	HCO_3_	HPO_4_
		
Units	dS m^-1^		mmol L^-1^							
Veg.	1.8	5.5	1.6	7.7	2.5	1.4	9.7	2.9	0.2	0.9
Veg. EC+	2.4	5.5	2.1	10.3	3.3	1.9	12.9	3.9	0.3	1.2
Gen.	1.8	5.5	0.9	9.9	2.5	0.9	11.5	2.7	0.0	0.7
Gen. EC+	2.4	5.5	1.3	13.1	3.3	1.1	15.3	3.5	0.0	0.9

	**Trace elements, potassium–calcium ratio, cation and anion equivalent sums**									
	
**Recipe**	**Fe**	**Mn**	**Zn**	**B**	**Cu**	**Mo**		**K/Ca**	**Anions**	**Cations**
			
**Units**	**μmol L^-1^**								**meq L^-1^**	

Veg.	67.5	11.3	5.6	22.5	1.1	1.1		3.5	16.9	16.7
Veg. EC+	65.5	10.9	5.5	21.8	1.1	1.1		3.4	22.6	22.3
Gen.	67.5	11.3	5.6	22.5	1.1	1.1		4.5	17.6	17.6
Gen. EC+	67.5	10.9	5.5	21.8	1.1	1.1		4.4	23.1	23.1

*The short day phase started after 18 (cycle 1), 20 (cycle 2) and 17 (cycle 3) days of long day phase.*

### Statistical Analysis

Harvests were on July 5 (cycle 1), November 14 (cycle 2) and February 10 (cycle 3). FW and DW of the aboveground parts, and plant height were determined as well as leaf number. Per treatment there were 2 (cycle 1), 2–6 (cycle 2), and 4 (cycle 3) replicate benches (**Table 4**). Each bench had an independently constructed and controlled irrigation system. Per bench 16 plants were randomly sampled, excluding guard plants along the bench sides. Values were averaged per bench as bench was the experimental unit. Data were analyzed as an unbalanced block design with cycles as blocks and model Cycle + Treatment. RUE was transformed to natural logarithmic values prior to analysis. Treatments were compared against all other treatments with pair wise *t*-tests at 5% (GENSTAT, Hemel Hempstead, UK).

**Table 4:** Aboveground growth parameters of *Chrysanthemum* as percentage of the value for DeepFlow_presspot_ and number of replicate benches per cycle.

		DeepFlow_presspot_	DeepFlow_water0_	DeepFlow_water1_	NutrientFlow	AeroPonic
Leaf Nr	no	100% a	86% c	87% bc	89% bc	90% b
Length	cm	100% a	91% c	91% c	92% bc	97% ab
FW	g	100% a	83% c	85% cb	94% abc	97% ab
DW	g	100% a	82% b^∗^	81% b	88% ab	94% ab^∗^
FW/CM	g.cm^-1^	100% a	91% a	93% a	102% a	100% a
RUE	g mol^-1^	100% a	85% b	83% b	92% ab	98% a

Cycle 1	rep.	2			2^∗∗^	2
Cycle 2	rep.	2	4		2	6
Cycle 3	rep.	4	4	4		4

*Data were averaged over three cultivation cycles with 16 plants per replicate bench. DeepFlow_*presspot*_, Deep Flow System with press pots, i.e., substrate-rooted cuttings. DeepFlow_*water0*_, Deep Flow System with water-rooted cuttings immersed in the nutrient solution surface. DeepFlow_*water1*_, Deep Flow System with water-rooted cuttings and the solution surface at 1 cm below the basal end of the cuttings. NutrientFlow, Nutrient Flow System; AeroPonic, Aeroponic Mist System. NutrientFlow and AeroPonic both used water-rooted cuttings. Leaf Nr, Number of leaves per harvested flower stem. 100% stands for DeepFlow_*presspot*_ with 31.1 leaves. Length, length of the harvested flower stem. 100% stands for DeepFlow_*presspot*_ with 86.5 cm. FW, fresh weight of harvested flower stem. 100% stands for DeepFlow_*presspot*_ with 130.3 g. DW, dry weight of harvested flower stem. 100% stands for DeepFlow_*presspot*_ with 14.1 g. FW/CM, fresh weight per cm harvested flower stem. 100% stands for DeepFlow_*presspot*_ with 1.51 g cm^-*1*^. RUE, Radiation Use Efficiency per harvested flower stem. The RUE data were log-transformed with the natural logarithm to reduce correlation of the means with variance. For reporting the logarithmic values were transformed back. 100% stands for DeepFlow_*presspot*_ with 0.853 g mol^-1^ PAR. a,b,c, cells without letters in common differ significantly with *P* = 0.05. ^∗^The pairs wise *t*-tests of all treatments against one another are condensed in this table but showed an additional significant difference for DW between DeepFlow_*water0*_ and Aeroponic (*P* = 0.020). ^∗∗^Data of NutrientFlow, cycle 1, excluded from statistical analysis. rep., number of replicate benches. A replicate being an ebb-and-flow bench with 120 plants of which 16 were harvested for observation.*

### Oxygen and Redox Potential

Dissolved oxygen was measured in the systems with a hand-held fiber optic oxygen meter (Neofox, Ocean optic, Dunedin, FL, USA). Redox potential was measured in some systems with a hand-held redox meter (GPRT 1400, Greisinger electronic, Regenstauf, Germany). Redox potential measurements were performed across the bench and before and after a fertigation event by inserting the probe half way into the solution.

### Growth Comparison

To account for differences in propagation duration (14 and 5 days), plant density (400 and 1200 plt m^-2^) and supplementary light (108 and 180 μmol m^-2^ s^-1^ PAR from high-pressure sodium lamps) growth comparisons were based on growth per unit radiation received using Radiation Use Efficiency (RUE). RUE, was defined as plant DW at the end of the cultivation in gram per plant divided by the incident radiation sum in mol PAR per plant, including solar and supplementary light (Lee et al., 2003). Solar light sums per day were calculated using the nearby Bleiswijk weather station 5-min data and the black-out screen settings. The solar radiation was taken to include 45% PAR of which 70% was transmitted into the greenhouse as incident PAR. The incident PAR was expressed per plant and then summed over the propagation and cultivation period. Market quality was evaluated using the auction criteria of a minimum stem length of 80 cm combined with a minimum weight per unit stem length of 1.0 g per cm.

## Results

### Yield Parameters and Radiation Use Efficiency

RUE, FW, and DW of DeepFlow_presspot_ were consistently the highest of the five treatments (**Table 4**). Both DeepFlow_water0_ and DeepFlow_water1_ had considerably lower RUEs than DeepFlow_presspot_; for DeepFlow_water0_ 85%; for DeepFlow_water1_ 83%. The plant DW compared to the plant DW in DeepFlow_presspot_ showed a similar pattern; DeepFlow_water0_ 82%; DeepFlow_water1_ 81%. The standard error for DeepFlow_presspot_ on DW in the second and third crop cycle was about twice that of the DeepFlow_water0_ and AeroPonic (additional data). Twenty percentage of the press pots held <30%-v/v of water immediately after transplanting because it took several days before roots crossed the 1–2 cm air gap between the press pot bottom and the solution surface. In contrast another 20% of the press pots held >70%-v/v of water when three or more roots between pot and nutrient solution surface were pulled together by the capillary force of water and acted as a wick.

The NutrientFlow in the crop cycle 2 had a RUE of 92% and a DW of 88% compared to DeepFlow_presspot_ (**Table 4**). Plant growth in crop cycle 1 was left out of the comparisons of treatments as evidence of zinc toxicity was found with zinc levels of 116 ± 7 mmol L^-1^ zinc between May 05, 2010 and June 08, 2010. In crop cycle 2 the cause, galvanized frames, was effectively isolated from the solution.

The AeroPonic performed as well as DeepFlow_presspot_ with a RUE of 97% compared to DeepFlow_presspot_ (**Table 4**).

The auction weight per cm stem and length criteria were met for all systems except NutrientFlow in crop cycle 1 and DeepFlow_water0_ and DeepFlow_water1_ which all three failed to meet the 80 cm length requirement.

The RUE data were log-transformed with the natural logarithm to reduce correlation of the means with variance. This improved the discrimination between the treatments slightly. RUE data show DeepFlow_presspot_ and AeroPonic to be equally efficient while DeepFlow_water0_ and DeepFlow_water1_ have statistically significant produced less efficient. NutrientFlow RUE data are in the middle of the range for the treatments and there is no statistically significant difference with any of the other treatments.

### Irrigation Supply

For DeepFlow_presspot_, the actual supply of water surpassed the maximum plant uptake almost ten times with an actual supply of 6.7 L m^-2^ h^-1^ and a maximum plant uptake of 0.7 L m^-2^ h^-1^ (**Table 5**). The actual supply of nutrients surpassed the maximum plant uptake about 16 times with an actual supply of 160 meq m^-2^ h^-1^ and a maximum plant uptake of 10 meq m^-2^ h^-1^ (**Table 5**). The actual supply of oxygen, however, was over 30 times lower than the maximum plant uptake of oxygen with an actual supply of 1.7 mmol m^-2^ h^-1^ and a maximum plant uptake of 63 mmol m^-2^ h^-1^ (disregarding the oxygen supply by solution mass flow in between irrigation cycles).

**Table 5:** Supply rates and maximum plant uptakes.

Parameter	Unit	DeepFlow_presspot_	DeepFlow_water0_	DeepFlow_water1_	NutrientFlow^1^	NutrientFlow^2^	AeroPonic
Water							
Supply^3^	L m^-2^ h^-1^	6.7	6.7	6.7	0.7	0.10^2^	40
Uptake^4^	L m^-2^ h^-1^	0.7	0.7	0.7	0.7	0.10^2^	0.7
EC							
Supply^3^	dS m^-1^	2.4	2.4	2.4	2.4	2.4	2.4
Supply^3,5^	meq m^-2^ h^-1^	160	160	160	16.8	2.4^2^	960
Uptake^4,5^	meq m^-2^ h^-1^	10	10	10	10	1.4^2^	10
Oxygen							
Supply^3^	mg L^-1^	8.0	8.0	8.0	8.0	8.0	8.0
Supply^3^	mmol L^-1^	0.3	0.3	0.3	0.3	0.3	0.3
Supply^3^	mmol m^-2^ h^-1^	1.7	1.7	1.7	0.2	0.025^2^	10
Uptake^4^	mmol m^-2^ h^-1^	63	63	63	63	8.9^2^	63

*Values are given per system, for water, nutrients, and oxygen expressed in the common units and when necessary converted to the equivalent per m^*–2*^ h^*–1*^ to allow comparing supply and plant uptake. For water, nutrients, and oxygen it is assumed all supply is through irrigation water supply only.*

*^*1*^Data on the NFT system on a m^*–2*^ basis so comparison with other columns is straight forward.*

*^*2*^Data on the NFT system on a m^*–1*^ basis so data correspond to the set-up with a 1 m long channel holding 10 plants.*

*^*3*^Supply stands for the supply rate of either water or nutrients or oxygen.*

*^*4*^Uptake stands for the maximum value for plant uptake rate of either water or nutrients or oxygen.*

*^*5*^Using milli-equivalents (millimol of cationic or anionic charge) to compare, assuming 1.0 dS m^*–1*^ to be equivalent to 10 meq anions (or 10 meq cations).*

**FIGURE 2:**
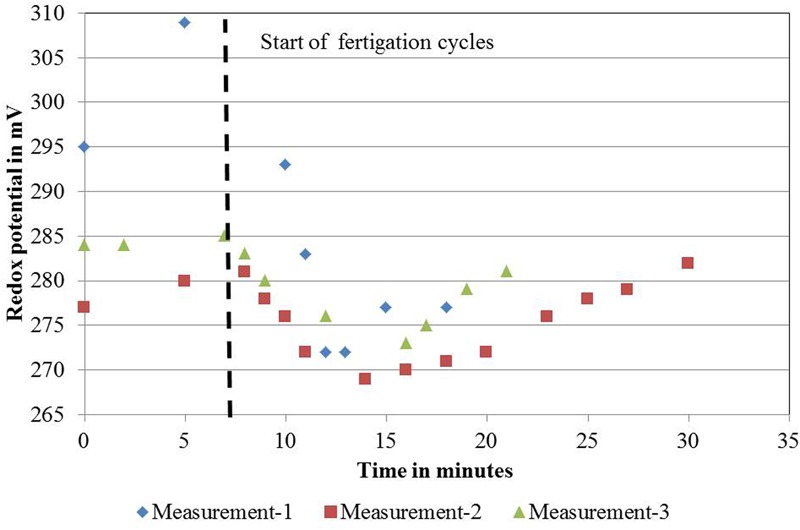
**Redox measurements on November 22, 2011 for one ebb-and-flow bench of the DeepFlow_presspot_.** DeepFlow_presspot_, Deep Flow System with press pots, i.e., substrate-rooted cuttings. Redox values in mV, duration of the measurements in minutes, starting 7 min before and lasting for 11–23 min after a fertigation cycle start (indicated by the vertical dotted line). Fertigation cycles started on 11:32 h for the first cycle; 12:02 for the second cycle and 12:35 for the third cycle.

For DeepFlow_water0_ and DeepFlow_water1_, the same supply rates for water, nutrients, and oxygen as for DeepFlow_presspot_ were realized. Redox measurements showed a decrease in potential from +305 to +270 mV, from nutrient solution inlet to the back of a bench as well as a rapid drop and slower increase in potential after a fertigation cycle, with +270 and +310 mV as the extremes (**Figure 2**). Oxygen measurements in solution were well above 5 mg L^-1^ with an average of 8.5 mg L^-1^ for DeepFlow_water1_ and 7.7 mg L^-1^ for DeepFlow_water0_.

For NutrientFlow, the actual water supply was equal to the maximum plant uptake, with an actual supply per row of 0.1 L m^-1^ h^-1^ and a maximum plant uptake of 0.10 L m^-1^ h^-1^ (**Table 5**). The actual nutrient supply just surpassed the maximum plant uptake with an actual supply per row of 2.4 meq m^-1^ h^-1^ and a maximum plant uptake of 1.4 meq m^-1^ h^-1^. The actual supply of oxygen, however, was almost 400 times lower than the maximum plant uptake of oxygen with an actual supply per row of 0.025 mmol m^-1^ h^-1^ and a maximum plant uptake of 8.9 mmol m^-1^ h^-1^ (disregarding the oxygen supply by solution mass flow in between irrigation cycles).

For AeroPonic the actual supply of water surpassed the maximum plant uptake almost sixty times with an actual supply of 40 L m^-2^ h^-1^ and a maximum plant uptake of 0.7 L m^-2^ h^-1^ (**Table 5**). The actual nutrient supply surpassed the maximum plant uptake about 100 times with an actual supply of 960 meq m^-2^ h^-1^ and a maximum plant uptake of 10 meq m^-2^ h^-1^. Oxygen supply through air was thought to be ample even though the water borne supply of oxygen was over six times lower than the maximum plant uptake of oxygen with an actual supply of 10 mmol m^-2^ h^-1^ and a maximum plant uptake of 63 mmol m^-2^ h^-1^.

### Nutrient Solution

Temporarily yellow leaves and permanently reddish brown leaf spots were frequently observed in the first 2 weeks of the cultivation phase, i.e., after transplanting in all systems and somewhat more in AeroPonic. In DeepFlow and AeroPonic low levels of iron and manganese were incidentally observed in some but not all replicates.

In cycle 1, in the storage tanks, a gradual drop of the nitrate level from 14 to 7 mmol L^-1^ was noticed over a period of 14 days before it was effectively countered by supplying a solution with EC 2.4 instead of 1.8 dS m^-1^. In cycle 2 storage tank pH dropped over 1.5 units under the targeted pH of 5.5 within a week after planting and before it was corrected. At the same time the ammonium level in the storage tank dropped from 1.5 to below 0.75 mmol L^-1^.

### Diseases

The diseases *Pythium* and sometimes *Fusarium* were present in “moderate” to “high” risk levels as witnessed by DNA fingerprint tests (additional data) in all tested systems but without visible root problems or above ground shoot symptoms. A high disease risk level could roughly corresponds to quantities of >10^-4^ colony-forming units, CFU.

### Rooting

**FIGURE 3:**
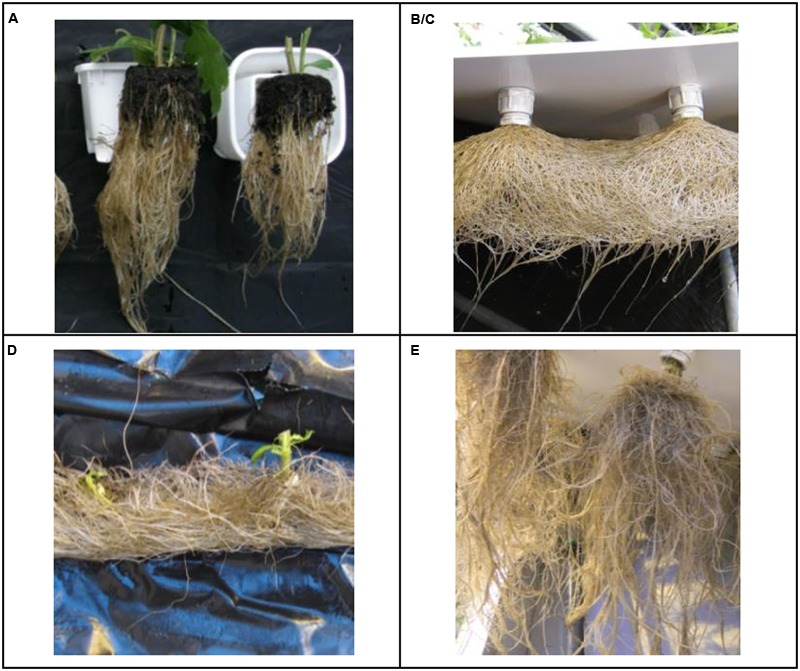
**Roots of mature plants as they grew in soilless systems.** DeepFlow_presspot_, Deep Flow System with press pots, i.e., substrate-rooted cuttings **(A)**. DeepFlow_water0_, Deep Flow System with water-rooted cuttings immersed in the nutrient solution surface **(B/C)**. DeepFlow_water1_, Deep Flow System, with water-rooted cuttings solution surface at 1 cm below the basal end of the cuttings **(B/C)**. NutrientFlow, Nutrient Flow System **(D)**. AeroPonic, Aeroponic Mist System **(E)**. NutrientFlow and AeroPonic both used water-rooted cuttings.

Fresh weight per cutting increased from 1.0 to 1.5 g in water based propagation and to 2.8 g in substrate based propagation. When we compared root growth patterns (**Figure 3**), we observed that NutrientFlow and DeepFlow_water0_ had tangled root mats which gradually turned brownish. Non-tangled roots in the DeepFlow and AeroPonic kept white roots longer. For the NutrientFlow treatment, in both crop cycles, the plant holes in the foil offered insufficient support to hold *chrysanthemum* cuttings upright. The lack of support caused plantlets to tilt out of the thin layer of nutrient solution flowing inside the channel. Tilting was corrected by hand but some plants in the guard row were left unchecked and most of the guard row tilted plants eventually wilted and died.

## Discussion

Both hypotheses were rejected: production on the water-based growing systems was not similar and production in the water-based cultivation systems was not higher when using cuttings from water-based propagation than when using cuttings from substrate-based propagation.

### DeepFlow Yield Performance and Transport Rates

Water-rooted cuttings in DeepFlow underperform when compared to substrate rooted cuttings in DeepFlow. Only 3% of the on average 19% lower DW production is explained by a lower radiation sum received per plant in the propagation of water-rooted cuttings (additional data). The remaining on average 16% lower RUE production of DeepFlow systems with water-rooted cuttings compared to substrate rooted cuttings may be explained by: (1) An oxygen transport rate to the upper root zone which limits plant growth (Morard et al., 2004; Kratky, 2005). However, the air gap introduced between cutting and nutrient solution in the DeepFlow_water1_ did not improve crop performance relative to DeepFlow_water0_. Therefore oxygen transport rate to the upper root zone does not explain the observed RUE differences. (2) A too low irrigation supply of oxygen to the lower root zone (Morard and Silvestre, 1996; Nakano, 2007). However, there is no reason why irrigation supply of oxygen to the lower root zone would affect growth different in the DeepFlow_presspot_ system than in the DeepFlow_water0_ system. Also the oxygen levels found in all DeepFlow are well above 5 mg L^-1^ which is thought ample for undisturbed growth (Soffer et al., 1991). Therefore oxygen transport rate to the lower root zone does not explain the observed RUE differences. (3) Hypoxia on a microscale within 1–2 mm from the root surface in dense root layers (Shi et al., 2007; Evans et al., 2009; Dresbøll and Thorup-Kristensen, 2012; Dresbøll et al., 2013). Such microscale hypoxia could easily occur in the root mat at the bottom of the comparatively shallow 4–6 cm deep DFT systems used here (**Figure 3**) rather than in the common DFT systems with 15–30 cm water in which roots are better distributed in the solution. But there seems no reason why hypoxic spots would affect growth different in the DeepFlow_presspot_ system than in the DeepFlow_water0_ system. So no convincing evidence of low oxygen availability on growth has been found.

An inherent drawback of the DeepFlow_presspot_ is the high density of press pots which is known to slow down growth (Morard and Silvestre, 1996; Evans et al., 2009; Dresbøll, 2010; Arancon et al., 2015) and which is reflected in the 30% increase in *chrysanthemum* weight inhomogeneity (**Table 4**, especially third crop cycle). Inhomogeneity is also increased by occasional capillary bridging of the *chrysanthemums* dense parallel roots. Admittedly DeepFlow_presspot_ like systems are used in practice, especially for lettuce growing (Nakano, 2007). Lettuce will, however, be less sensitive to capillary bridging of roots because of its pronounced tap root.

### NutrientFlow Yield Performance and Transport Rates

It was hypothesized NFT would outperform DeepFlow_presspot_ but the RUE yield level in crop cycle 2 was 8% below the DeepFlow_presspot_ RUE. We explain part of the difference with the actual NFT irrigation settings. (1) For water the supply was 30% lower than required to meet the maximum plant uptake as calculated. The practical consequences are still limited as the number of hours the water supply was inadequate is about 10 h over the whole cropping period, i.e., relatively minor. (2) For nutrients, the supply seemed just adequate to meet the maximum plant uptake but as will be discussed under “nutrients,” the actual plant uptake was double the prior calculated maximum plant uptake, which makes it likely the supply of nutrients has been sub optimal for several hours on most days. Suboptimal nutrient supply would also be in line with the observed lowest DW over FW ratio of all treatments. We found no plant weight depression caused by depletion of the nutrient solution along the length of the gullies used as is reported for much longer NFT units (Gislerød and Kempton, 1983). (3) For oxygen we calculated a maximum plant uptake of 8.9 mmol m^-1^ h^-1^ for a 10 plant row based on prior assumptions (Morard et al., 2000; Bar-Yosef and Lieth, 2013; Blok and Gérard, 2013) which is about 350 times higher than the steady 0.025 mmol m^-1^ h^-1^ irrigation supply realized. However, mass transport of oxygen from the surrounding air in the meandering and 1–5 mm shallow flow may supply a large part of the required oxygen (Gislerød and Kempton, 1983).

Therefore the irrigation supply of water, nutrients, and oxygen was too low to meet the maximum plant uptake as estimated and especially nutrient supply may have limited crop performance.

### AeroPonic Yield Performance and Transport Rates

The AeroPonic RUE yield level was 2% below the DeepFlow_presspot_. As we used 40 L m^-2^ h^-1^ irrigation supply (**Table 5**) we amply met maximum plant uptake for water (0.7 L m^-2^ h^-1^) and nutrients (10 meq m^-2^ h^-1^). On a plant to plant scale, however, this may be different because when root lengths increase, plants positioned further away from the emitters will be shielded and will receive a lot less water and nutrients. The multi-point supply therefore requires an unknown but considerable overcapacity in supply to ensure all plant positions receive enough water and nutrients to meet the maximum plant uptake. The irrigation supply did not meet the maximum plant uptake for oxygen (63 mmol m^-2^ h^-1^), however, the oxygen supply rate in the thin layer of nutrient solution on the roots was assumed to be not limiting as roots were hanging in air with 21%-w/w of oxygen at all times.

Because the irrigation supply of water, nutrients, and mostly air derived oxygen in AeroPonic was thought to be ample to meet plant uptake, it was unexpected to find nutrient deficiency symptoms for all systems but specifically AeroPonic in the first 5–10 days after transplanting. For AeroPonic transient nutrient depletion in between fertigation cycles on the root surface has been reported (Ingestad and Ågren, 1988). This, again, calls for measurements on microscale on the root surface. An alternative explanation is the presence of a sheet of root based microbial life, either competing with or physically hindering root uptake.

### Transport Rates

System related supply rates for water, nutrients, and oxygen were compared to maximum plant uptakes based on literature. Some limitations of using maximum plant uptakes are: (1) For plant water use, the assumed maximum of 0.7 L m^-2^ h^-1^ is valid only for the defined radiation level. Greenhouses with more natural light and or extreme artificial light supply may show a higher transpiration level as will greenhouses with more transparent decks. Furthermore if there is a high flow of hot dry air, transpiration may show a higher transpiration level (Steppe et al., 2008; Vermeulen et al., 2012; Janka et al., 2016). (2) For plant nutrient use, we assumed non-limiting carbon dioxide supply. The maximum of 10 meq m^-2^ h^-1^ is furthermore valid only for common growth. Over-consumption after a period of starving or when nutrients precipitate in the plant as for calcium oxalate, will increase the uptake. The uptake is also increasing over the years, as newer varieties have higher production rates and growth conditions in commercial greenhouses keep on being improved (Higashide and Heuvelink, 2009). Nevertheless, the relation of uptake with light use efficiency or light level is extensively used in models (Mankin and Fynn, 1996; Gorbe and Calatayud, 2010; Signore et al., 2016). (3) For plant oxygen use the oxygen supply in the amount of water needed to compensate plant use is almost always insufficient (Ehret et al., 2010; Dorais and Pepin, 2011). Systems must be evaluated taking into account that oxygen is supplied mainly by mass flow and some diffusion. It is still very difficult to describe mass flow supply on micro scale.

Lower maximum uptake rates are to be expected with any sub-optimal growth condition of which we only mention the absence of carbon dioxide supply and sub optimal light interception by the crop as likely causes.

### Nutrients

Nutrient solutions for water-based growing required several adaptations: (1) Iron and manganese levels were doubled to compensate for precipitation by oxidation in the aerated solutions. (2) Ammonium was left out of the start solution to prevent bacterial acidification caused by plant and bacterial luxury ammonium consumption (Sonneveld and Voogt, 2009; Horchani et al., 2010; Dickson et al., 2016). (3) The EC was increased from 1.8 to 2.4 dS m^-1^ to avoid depletion of nitrate and, to a lesser extent, calcium and potassium. The nitrate depletion itself may indicate an increased plant growth compared to the growth the original recipe was based upon, but more importantly it proves the plant uptake concentration, used to calculate maximum plant uptake, was too low with 1.0 L with EC 1.0 (which is 10 meq m^-2^ h^-1^). The observed uptake EC was higher than 1.8 but lower than 2.4 dS m^-1^. We now assume a plant uptake EC of at least 2.0 and possibly 2.2 over a period of several weeks, in line with unpublished results for a commercial tomato crop (additional data). This would mean that the peak requirement for nutrients is easily 22 meq m^-2^ h^-1^ but possibly as high as 30 meq m ^-2^ h^-1^. This indicates the sum of hours that the irrigation supply of nutrients could not meet the plant uptake, must have been well above 10 h over the cultivation period including contiguous periods of several hours on bright days. Once plant uptake is higher than the transport rate toward the roots, serious depletion can result within half an hour (Aguilar et al., 2003).

### Diseases and Volumes

Disinfection costs will increase with increasing resident water volumes of the system, favoring NutrientFlow and AeroPonic over the DeepFlow variants (**Table 2**). The smaller resident water volumes allow the use of UV sterilization to check water borne diseases. Up to now growers use water-based growing without sterilization. There is, however, no reason why disinfection, which is a prerequisite in substrate-based growing, should not be implemented in water-based growing. Moreover, our incidental DNA checks on diseases showed levels of *Pythium* and *Fusarium* high enough to call for disinfection in all water-based growing systems. Water-based growing systems with resident diseases are very sensitive to lack of oxygen derived *Pythium* infection (Cherif et al., 1997), typically followed by *Phytophthora* and *Fusarium*.

### Rooting

The discoloration of the matted roots in NutrientFlow and DeepFlow_water0_ was most likely the result of an at times too slow transport rate of oxygen into the root mat. The maximum plant uptake for oxygen was calculated to require 63 mmol m^-2^ h^-1^ which would require a saturated water flow of 250 L m^-2^ h^-1^. Oxygen is, however, not only supplied by the irrigation water, but also by diffusion and mass flows (Jungk, 2002; Silber et al., 2003). Diffusion in water is very slow and not of practical consequence. Mass flows are unknown but potentially large enough. Mass flows may be caused by pumps and temperature gradients, which allows water to partly re-saturate with oxygen at the nutrient solution surface and to carry that oxygen to the roots (Jungk, 2002). Therefore the irrigation supply will not have to meet the total plant uptake as long as mass flow will supply most of the requirement. When roots start to intertwine and shield each other, however, local flows are reduced with orders of magnitude. This means the transport rate of oxygen to dense root layers, as in the DeepFlow systems used, can be limiting despite large flows in the surrounding nutrient solution.

### Transplants

It was originally hypothesized that transplanting into the same medium, in this case from propagation solution into cultivation solution, might overcome the 3–5 days growth reduction on transplanting (Arancon et al., 2015). This growth reduction is attributed to (a) regrowth of roots into the new medium (b) establishment of a new root surface population of microorganisms (Jack et al., 2011). Instead, we observed plants transplanted from nutrient solution into nutrient solution still struggled for about 14 days to establish themselves while showing signs of transient discoloration. Assuming a growth-reducing presence of micro-organisms on the root surface, it could be of interest to check on their presence, diversity, and nature (Jack et al., 2011) and to check on the exchange of gasses other than oxygen (Zhao et al., 2010). Future research might also explore the optimum combination of duration, plant density, and light level in propagation to possibly boost the RUE results of water-based growing.

### Economics

Economics of the soilless systems were studied (van Os et al., 1991; Hansen, 1999; Blok and Vermeulen, 2012). They concluded investments in soilless systems require a 10–15% yield increase over soil grown *chrysanthemum* to become feasible. In this study, we used scale adapted commercially available systems for DFT (lettuce system) and NFT (lettuce, tomato, strawberry, and *chrysanthemum*). The aeroponics system was not yet marketed. For all water-based systems the water flow must be high enough at least meet the plant use but not much higher in order to control costs for pumping and disinfection. Year round water use efficiencies of all systems used were estimated to be 7.0 g/L for dry mass production and 57 g/L for fresh mass production which is high compared to soil grown *chrysanthemum* in which there is at least 30–50% drainage loss as well as an estimated 10% lower mass production.

## Conclusion

Plant production in completely water-based systems did not outperform a system combining substrate-based propagation and water-based cultivation. The actual irrigation supply for water, nutrients, and oxygen in all water-based systems was often below the maximum plant uptake for water, nutrients, and oxygen. This means plant uptakes for water, nutrients, and oxygen were not at all times met by irrigation rate and distribution in water-based cultivation systems. Plant uptake rates for water, nutrients, and oxygen are offered as a more fundamental way to compare growing systems. The plant uptake rates can also be the basis for a design check and possible redesign of water-based systems. At the same time flows must be minimized to reduce costs for pumping and disinfection. Crop performance expressed as RUE was highest for the reference system, DeepFlow_presspot_ and the AeroPonic (-2%). The NutrientFlow RUE yield was not significantly lower (-8%) and the RUE yield on the DeepFlow_water0_ and DeepFlow_water1_ were significantly lower than the DeepFlow_presspot_ and the AeroPonic (-15% on average).

## Author Contributions

CB conceived and designed the experiments; XG performed the experiments; BJ, PdV, LM, and CB discussed and wrote the main body of the paper.

## Conflict of Interest Statement

The authors declare that the research was conducted in the absence of any commercial or financial relationships that could be construed as a potential conflict of interest.
